# Supplementary material for: Decreased Levels of Microfibril-Associated Glycoprotein (MAGP)-1 in Patients with Colon Cancer and Obesity Are Associated with Changes in Extracellular Matrix Remodelling
Source: Int J Mol Sci. 2021 Aug 6;22(16):8485. doi: 10.3390/ijms22168485 (PMC8395192; doi:10.3390/ijms22168485)
Supplement: Supplementary file 1 [file ijms-22-08485-s001.zip › ijms-1291934-supplementary.pdf]

Supplemental Table S1. Pathological characteristics of patients with colon cancer.

**Location of primary lesion**	**n**
Transverse colon	5
Right hemicolon	13
Left hemicolon	15
Missing	3
**TNM stage**	**n**
I	5
II	7
III	17
IV	4
Missing	3
**Differentiation**	**n**
Well	3
Moderately	25
Poorly and undifferentiated	5
Missing	3
**Tumor size**	**n**
≤ 5 cm	17
> 5 cm	8
Missing	11
**Lymph node status**	**n**
Positive	12
Negative	23
Missing	1

TNM, tumor, nodes, metastasis.

Supplemental Table S2. Primers and probes sequences.

Gene (GeneBank accession)	Sequences
COL6A3 (NM_004369.3)	
Forward	GACGGAGATCTGGCTGATTTACA
Reverse	AGATGCATTAGCCGCTCCAA
TaqMan® Probe	FAM-AGAACCTCCGCCAAGAAGGAGTCCGT-TAMRA
CTNNB1 (NM_001098209.2)	
Forward	ACGGCCTGCAGAGCTATTCA
Reverse	TGATCTTCTGCATGTTCCCAAAC
TaqMan® Probe	FAM-ATTCCTCTCTGACGGCGTGCTCTTCAGT-TAMRA
DCN (NM_001920.5)	
Forward	AGAAGCTCTCCTACATCCGCATT
Reverse	CTGCATCAACTCTGCTGATTTGT
Probe	FAM-TTCCTCAAGGTCTTCCTCCTTCCCTTACG-TAMRA
MFAP2 (NM_002403)	
Forward	CAGTGTCTCAACGAGGTCTGCTT
Reverse	ATGGGCACACACTGTACGAACA
TaqMan® Probe	FAM-TACAGCCTCCGCCGTGTGTACGTCAT-TAMRA
MUC2 (NM_002457)	
Forward	ACGGCCTGCAGAGCTATTCA
Reverse	TGATCTTCTGCATGTTCCCAAAC
TaqMan® Probe	FAM-ATTCCTCTCTGACGGCGTGCTCTTCAGT-TAMRA
PTGS2 (NM_000963.4)	
Forward	GCTCAGCCATACAGCAAATCCT
Reverse	TGGTCAAATCCCACACTCATACATA
TaqMan® Probe	FAM-TGTTCCCACCCATGTCAAAACCGAGG-TAMRA
SPP1 (NM_000582)	
Forward	CATCCAGTACCCTGATGCTACAGA
Reverse	GGCCTTGTATGCACCATTCAA
TaqMan® Probe	FAM-ACATCACCTCACACATGGAAAGCGAGGA-TAMRA
TGFB1 (NM_000660)	
Forward	GCCCAGCATCTGCAAAGC
Reverse	TCCTTGCGGAAGTCAATGTACA
TaqMan® Probe	FAM-CACCAACTATTGCTTCAGCTCCACGGA-TAMRA

COL6A3, collagen type 6 α3 chain; *CTNNB1*, catenin β; *DCN*, decorin; *MFAP2*, microfibril-associated protein 2; *MUC2*, mucin 2; *PTGS2*, prostaglandin-endoperoxide synthase 2; *SPP1*, secreted phosphoprotein 1; *TGFB1*, transforming growth factor β.

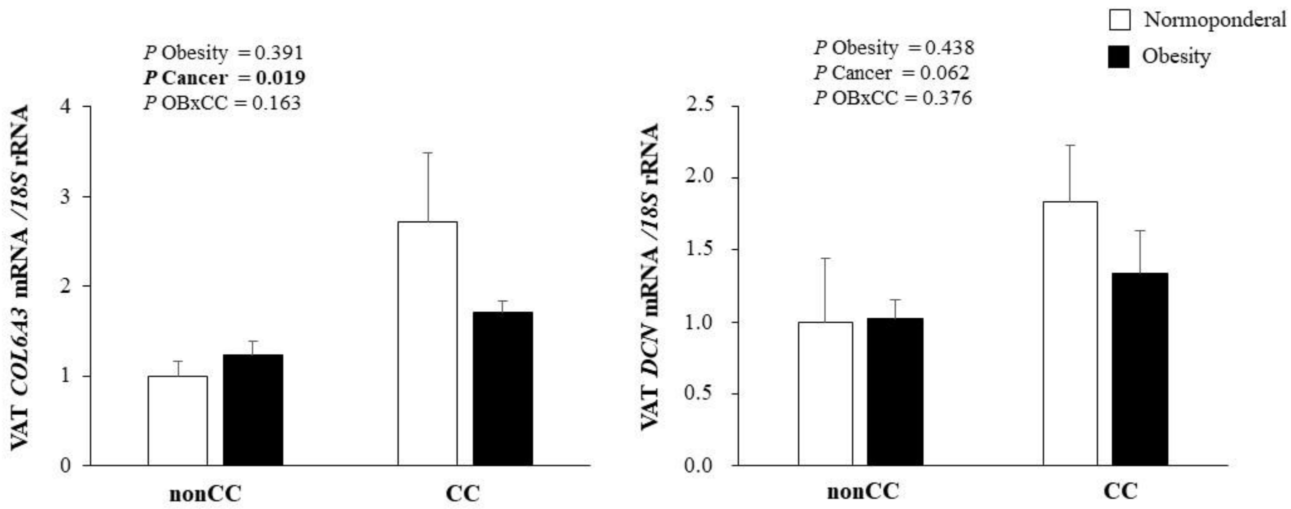

Supplemental Figure S1. Effect of obesity and colon cancer (CC) on visceral adipose tissue (VAT) gene expression levels of (A) *COL6A3* and (B) *DCN* from normoponderal subjects (NP) and volunteers with obesity (OB) classified according the presence or absence of colon cancer (CC). Bars represent the mean ± SEM. Differences between groups were analyzed by two-way ANCOVA. *COL6A3*, collagen type 6 α3 chain; *DCN*, decorin.

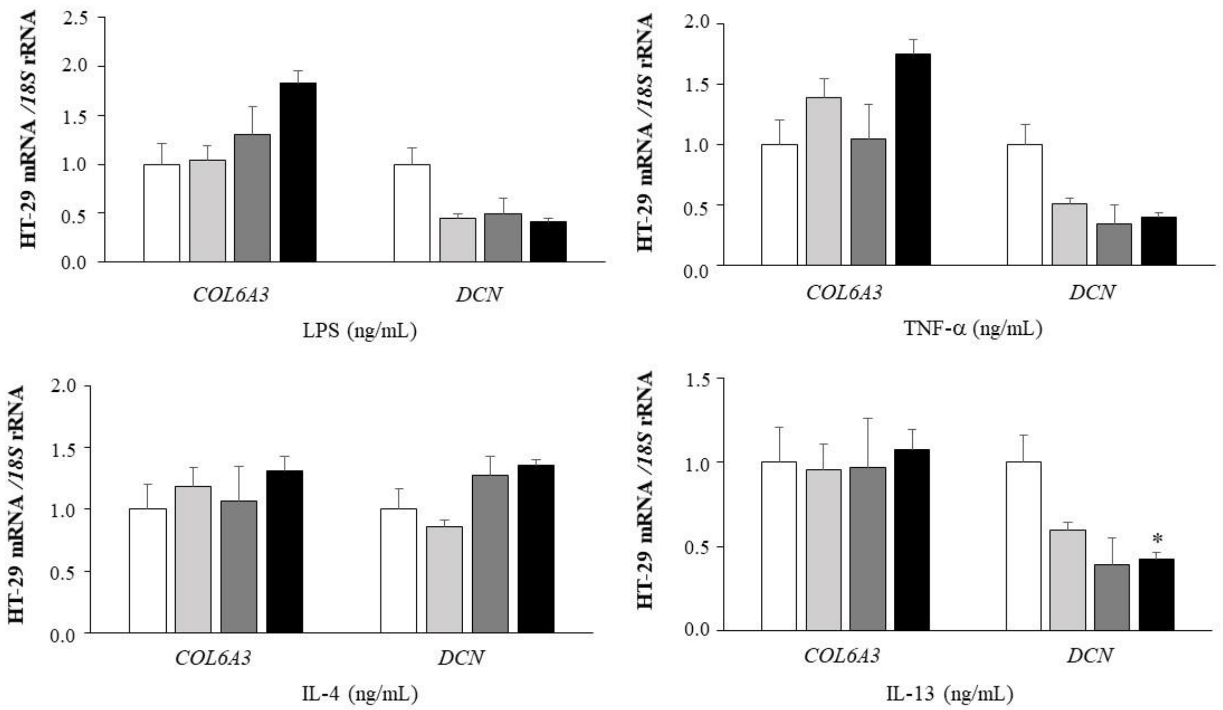

Supplemental Figure S2. Impact of inflammation-related factors in the gene expression levels of the *COL6A3* and *DCN* in HT-29 colon cancer cells. Bar graphs show the effect of 24-h treatment with (A) LPS, (B) TNF-α, (C) IL-4 and (D) IL-13 13 on *COL6A3* and *DCN* mRNA levels. Values are the mean ± SEM (n=6 per group). Differences between groups were analyzed by one-way ANOVA followed by Dunnett's tests. $^{*}P<0.05$ *vs* unstimulated cells. *COL6A3*, collagen type 6 α3 chain; *DCN*, decorin.
